# The mitochondrial protein Bcs1A regulates antifungal drug tolerance by affecting efflux pump expression in the filamentous pathogenic fungus *Aspergillus fumigatus*

**DOI:** 10.1128/spectrum.01172-24

**Published:** 2024-08-20

**Authors:** Guorong Yang, Weiwei Shi, Wenlin He, Jing Wu, Sutao Huang, Li Mo, Junjie Zhang, Huaxue Wang, Xiaogang Zhou

**Affiliations:** 1 Anhui Key Laboratory of Infection and Immunity, School of Basic Medicine, Bengbu Medical University, Bengbu, China; 2 Departments of Critical Care Medicine, The First Affiliated Hospital of Bengbu Medical University, Bengbu, China; 3 School of Life Sciences, Bengbu Medical University, Bengbu, China; 4 School of Fundamental Sciences, Bengbu Medical University, Bengbu, China; Universidade de Sao Paulo, Ribeirao Preto, Sao Paulo, Brazil

**Keywords:** *Aspergillus fumigatus*, Bcs1A, mitochondria, drug resistance, vegetative growth

## Abstract

*Aspergillus fumigatus* is the predominant pathogen responsible for aspergillosis infections, with emerging drug-resistant strains complicating treatment strategies. The role of mitochondrial functionality in fungal resistance to antifungal agents is well-documented yet not fully understood. In this study, the mitochondrial protein Bcs1A, a homolog of yeast Bcs1, was found to regulate colony growth, ion homeostasis, and the response to antifungal drugs in *A. fumigatus*. Microscopic observations revealed substantial colocalization of Bcs1A-GFP fusion protein fluorescence with mitochondria. Bcs1A deletion compromised colony growth and the utilization of non-fermentable carbon sources, alongside causing abnormal mitochondrial membrane potential and reduced reactive oxygen species production. These findings underscore Bcs1A’s vital role in maintaining mitochondrial integrity. Phenotypic analysis and determinations of minimum inhibitory concentrations indicated that the Δ*bcs1A* mutant was more resistant to various antifungal agents, such as azoles, terbinafine, and simvastatin, compared to wild-type strain. RNA sequencing and RT-qPCR analysis highlighted an upregulation of multiple efflux pumps in the Δ*bcs1A* mutant. Furthermore, loss of the principal drug efflux pump, mdr1, decreased azole tolerance in the Δbcs1A mutant, suggesting that Bcs1A’s modulated of azoles response via efflux pump expression. Collectively, these results establish Bcs1A as essential for growth and antifungal drug responsiveness in *A. fumigatus* mediated through mitochondrial regulation.

**IMPORTANCE:**

Drug resistance presents a formidable obstacle in the clinical management of aspergillosis. Mitochondria are integral to various biochemical pathways, including those involved in fungi drug response, making mitochondrial proteins promising therapeutic targets for drug therapy. This study confirms that Bcs1A, a mitochondrial respiratory chain protein, is indispensable for mitochondrial functionality and multidrug tolerance in *Aspergillus fumigatus*. Mutation of Bcs1A not only leads to a series of drug efflux pumps upregulated but also shows that loss of the primary efflux pump, *mdr1*, partial reduction in drug tolerance in the Bcs1A mutant, highlighting that Bcs1A’s significant influence on mitochondria-mediated drug resistance.

## INTRODUCTION

Invasive aspergillosis represents a significant threat to human health, especially among immune-compromised individuals, leading to substantial high morbidity and mortality rates (1–3). *Aspergillus fumigatus*, a ubiquitous environmental saprotrophic fungus, is the principal causative agent of this disease (3–5). In 2022, the World Health Organization recognized *A. fumigatus* as a critical fungal pathogen affecting human health (6).

Clinically, aspergillosis is treated with various antifungal agents, including azoles, echinocandins, and polyenes (7–10). Azoles are often preferred due to their efficacy and lower side effects profile (11, 12). Nevertheless, the rise in azole-resistant strains, driven by their extensive use in both clinical and agricultural settings, poses a significant challenge in treating aspergillosis (13–17). The mechanisms of azole resistance have been extensively explored, focusing on mutations in the target protein, enhanced efflux pump expression, and activation of stress response pathways (18–22). Particularly, alterations in the *cyp51/erg11* gene have been identified as a major resistance factor (20, 23). However, recent data indicate a growing prevalence of mechanisms that do not involve cyp51, underscoring the urgency of understanding these novel resistance pathways to develop more effective therapeutic strategies(24).

The mitochondrion plays a multifaceted role in fungal cells, impacting lipid metabolism, ion homeostasis, and cell wall integrity (25–27). Research has highlighted a potential link between mitochondrial function and antifungal resistance (28, 29). For instance, in yeast, mitochondrial DNA (mtDNA) deletions have been associated with increased azole resistance (30, 31). Moreover, fungal mitochondria undergo continuous fission and fusion to increase adaptability (32–34). In the pathogenic fungus *Candida albicans*, mutations in the Fzo1 gene lead to defective mitochondrial fusion and increase fungus susceptibility to azole (35). Conversely, the deletion of mitochondrial fission genes (Δ*fis1*, Δ*dnm1*, and Δ*mdv1*) in the filamentous pathogenic fungus *A. fumigatus* can significantly increase resistance to azole (36). Furthermore, mutations in heme biosynthetic pathway genes (*cox7*, *cox10*, and *cox15*) lead to mitochondrial dysfunction that contributes to drug resistance in both yeast and *A. fumigatus* (37–39). Likewise, in *Candida glabrata*, impaired mitochondrial respiration resulted in reduced sensitivity to azoles (40). Overall, maintaining optimal mitochondrial function is critical for drug resistance in fungi, suggesting that targeting mitochondrial proteins could be a promising avenue for developing novel antifungal drugs.

Mitochondrial oxidative phosphorylation (OXPHOS) is crucial for ATP production within the cell, relying on the assembly of five multi-subunit complexes (I–V) within the inner mitochondrial membrane (41). The assembly of these complexes involves subunits encoded both by mtDNA and nuclear DNA (41–44). Among these, Bcs1, a conserved protein containing ATPases associated with diverse cellular activities (AAA) domain, is integral to the assembly of the mitochondrial respiratory chain cytochrome *bc1* complex (45). Bcs1 facilitates the transport and insertion of the Rieske Fe/S protein, Rip1, into respiratory complex III (46–49). In yeast, an absence of Bcs1 results in an accumulation of Rip1 in the mitochondrial matrix and deficiencies in complex III (50, 51). Mutations in BCS1L, the human homolog of Bcs1, have been associated with several serious diseases (52). The Bcs1 is critical to hyphal growth and the maintenance of intracellular lipid homeostasis in *Beauveria bassiana* (53). Recent studies have confirmed the involvement of Bcs1 in the response to azole (54), although the precise underlying molecular mechanism is poorly understood.

In this study, we investigated the role of the mitochondrial protein Bcs1A in regulating various cellular processes such as growth, reactive oxygen species (ROS) production, maintenance of the mitochondrial membrane potential, ion homeostasis, and the multidrug response in *A. fumigatus*. Importantly, our findings demonstrated that the primary mechanism driving Bcs1A-mediated antifungal tolerance may be the upregulation of efflux pumps.

## RESULTS

### Bcs1A is the homolog of the yeast Bcs1 in *A. fumigatus*

**Fig 1:**
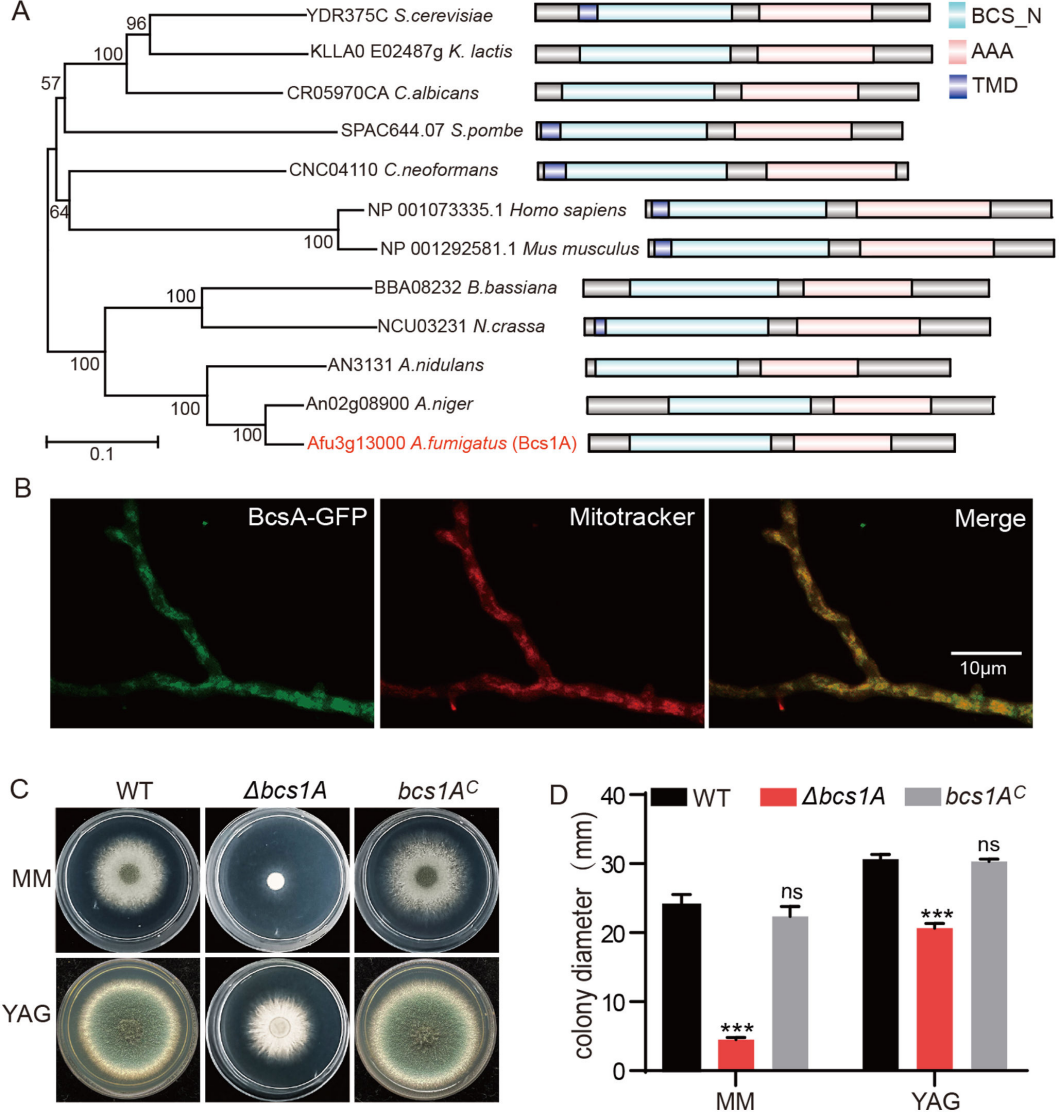
Mitochondria-localized Bcs1A is required for hyphal growth in *A. fumigatus*. (**A**) Bioinformatic analysis of selected Bcs1 homologs from eukaryotes. The phylogenetic tree was constructed using MEGA 7 software. Domains were analyzed using SMART (http://smart.embl-heidelberg.de/). (**B**) Subcellular localization of green fluorescent protein (GFP)-tagged Bcs1A. MitoTracker was used to visualize mitochondria. Scale bar, 10 µm. Colony phenotypes (**C**) and diameters (**D**) of the wild-type (WT), Δ*bcs1A*, and *bcs1A^C^* strains grown in minimal medium (MM) and yeast extract-glucose (YG) media at 37°C for 48 h. ns, not significant; ***, *P* < 0.001.

Homologs of the yeast mitochondrial electron transfer chain complex III protein Bcs1 (*bc1* synthesis) were explored by BLASTP searches in the Fungal Genome Database. The search identified a putative mitochondrial protein, Bcs1A (AFUA_3G13000), showing 46.95% identity and an E-value of 0.0, suggesting a significant similarity. Phylogenetic analyses of Bcs1 proteins from a diverse range of eukaryotes, from filamentous fungi to *Homo sapiens*, confirmed the presence of orthologs of Bcs1A. Notably, all selected Bcs1 homologs exhibited key domains including BCS1_N and AAA domains (Fig. 1A).

**Fig 2:**
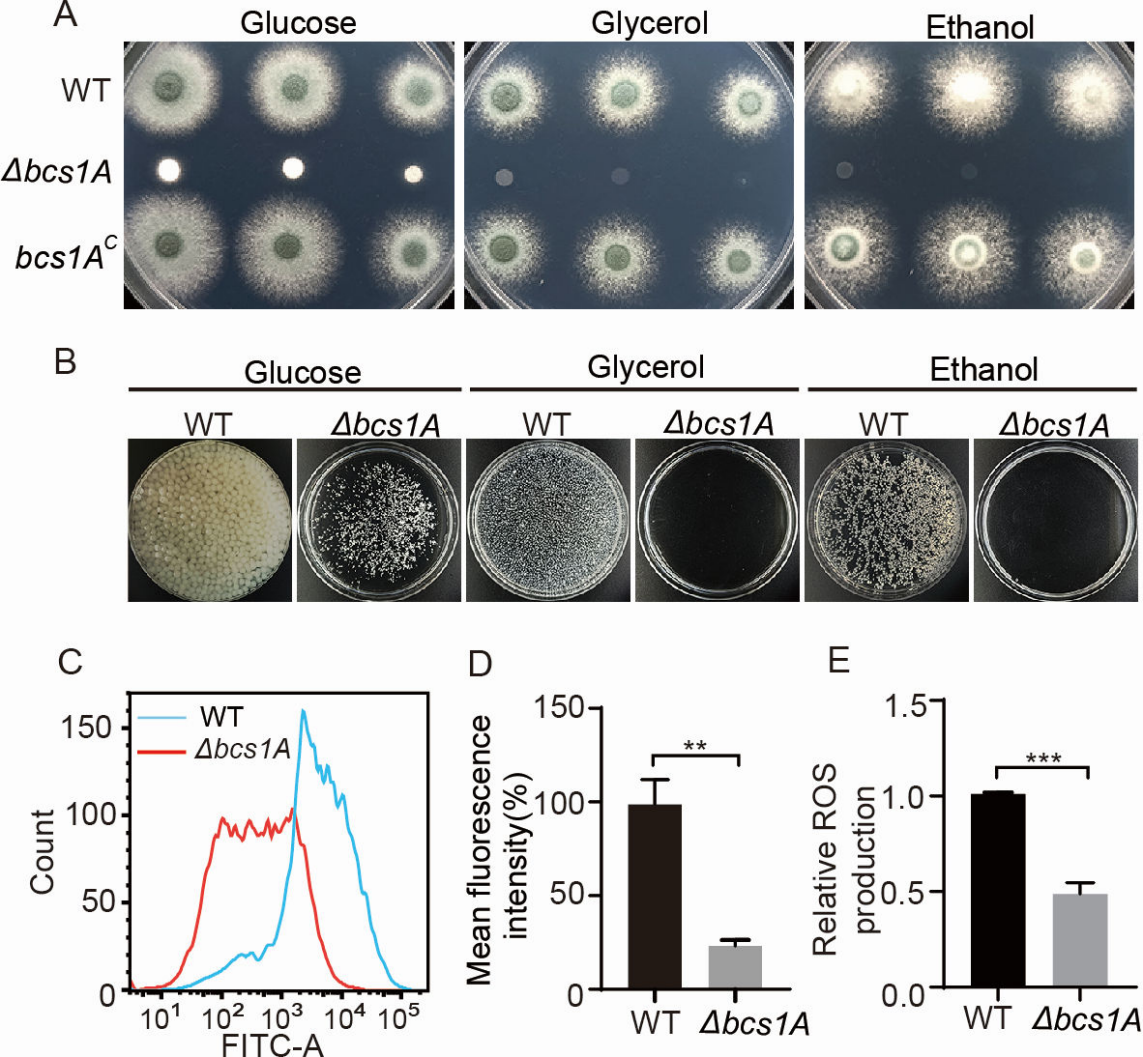
Bcs1A is essential for maintaining optimal mitochondrial function in *A. fumigatus*. (**A**) Colony morphologies of the WT, Δ*bcs1A,* and *bcs1A^C^* strains on solid medium containing glucose, glycerol, and ethanol as carbon sources, respectively, at 37°C for 48 h. (**B**) The indicated strains were cultured in a liquid medium supply with glucose, glycerol, and ethanol as carbon sources, respectively, with shaking at 37°C, 220 rpm for 48 h. (**C**) The mitochondrial membrane potential was assessed using flow cytometry, with FITC-A on the x-axis indicating the relative fluorescence intensity value. (**D**) Quantitative and statistical analyses of the mean fluorescence intensity and (**E**) ROS generation in the WT and Δ*bcs1A* mutant strains. **, *P* < 0.01; ***, *P* < 0.001.

To elucidate the function of Bcs1A, a green fluorescent protein (GFP)-tagged Bcs1A strain was created by the incorporation of GFP at the C-terminus of Bcs1A under the control of its natural promoter (Fig. S1A). The resultant strain displayed similar colony phenotypes compared to the wild-type (WT) strain (Fig. S1B), confirming the functionality of the GFP-tagged Bcs1A. Fluorescence microscopy images showed mitochondria expression of Bcs1A-GFP (Fig. 1B), analogous to ScBcs1 in yeast (47). Mitochondrial localization was further validated using MitoTracker Red CMXRos, which showed an overlap of red fluorescence with the GFP signal, clearly indicating the mitochondrial residence of Bcs1A (Fig. 2B).

To investigate the role of Bcs1A more deeply, a Δ*bcs1A* mutant strain lacking the full-length *bcs1A* gene was constructed through homologous recombination, substituting with the nutritional marker *pyrG* in the WT background (Fig. S2A). This deletion was verified using diagnostic PCR (Fig. S2B). Comparative growth analysis demonstrated that the Δ*bcs1A* mutant exhibited significant growth impairments on both minimal medium (MM) and yeast extract-glucose (YG) medium, in stark contrast to the WT strain (Fig. 1C and D). Conversely, the *bcs1A* complementation strain, where the Bcs1A gene was reintroduced, restored normal growth, paralleling the WT phenotype. These results underscore the crucial role of Bcs1A in supporting hyphal growth and overall vitality in *A. fumigatus*.

### Bcs1A is required for proper mitochondrial function

Previous research indicates that Bcs1 is a mitochondrial protein essential for the biogenesis of the respiratory chain complex III in eukaryotic organisms (45–47). Based on this understanding, it was hypothesized that Bcs1A plays a similar critical role in mitochondrial function within *A. fumigatus*. Mitochondria are pivotal for the proper metabolism of intracellular carbon sources. Notably, the *bcs1A* mutant displayed profound growth impairments when cultured on both solid (Fig. 2A) and liquid minimal media (Fig. 2B) supplemented with non-fermentable carbon sources such as glycerol or ethanol, compared to the parental WT and complemented strains. To further explore the mitochondrial function in these strains, rhodamine 123 (Rh123), a marker for mitochondrial membrane potential (MMP), was employed. Flow cytometry analysis revealed a significant reduction in mean fluorescence intensity in the Δ*bcs1A* mutant relative to the WT, indicating impaired mitochondrial function in the mutant (Fig. 2C and D). Additionally, the intracellular levels of mitochondria-derived ROS were assessed. The Δ*bcs1A* mutant exhibited decreased ROS production compared to the WT, further supporting the compromised mitochondrial function (Fig. 2E). Previous studies have also highlighted the role of mitochondria, as osmosensing organelles capable of directly responding to osmotic stress in eukaryotes (55). To test the osmotic sensitivity of the Δ*bcs1A* mutant, strains were grown on MM supplemented with osmotic stressors such as KCl, NaCl, or sorbitol. The results showed that the Δ*bcs1A* mutant was more susceptible to osmotic stress compared to the WT strain (Fig. S3). Collectively, these findings underscore the essential role of Bcs1A in maintaining mitochondrial function in *A. fumigatus*, affecting not only energy metabolism and ROS production but also the organism’s ability to respond to environmental stress.

### Bcs1A is required for the antifungal drug response in *A. fumigatus*

**Fig 3:**
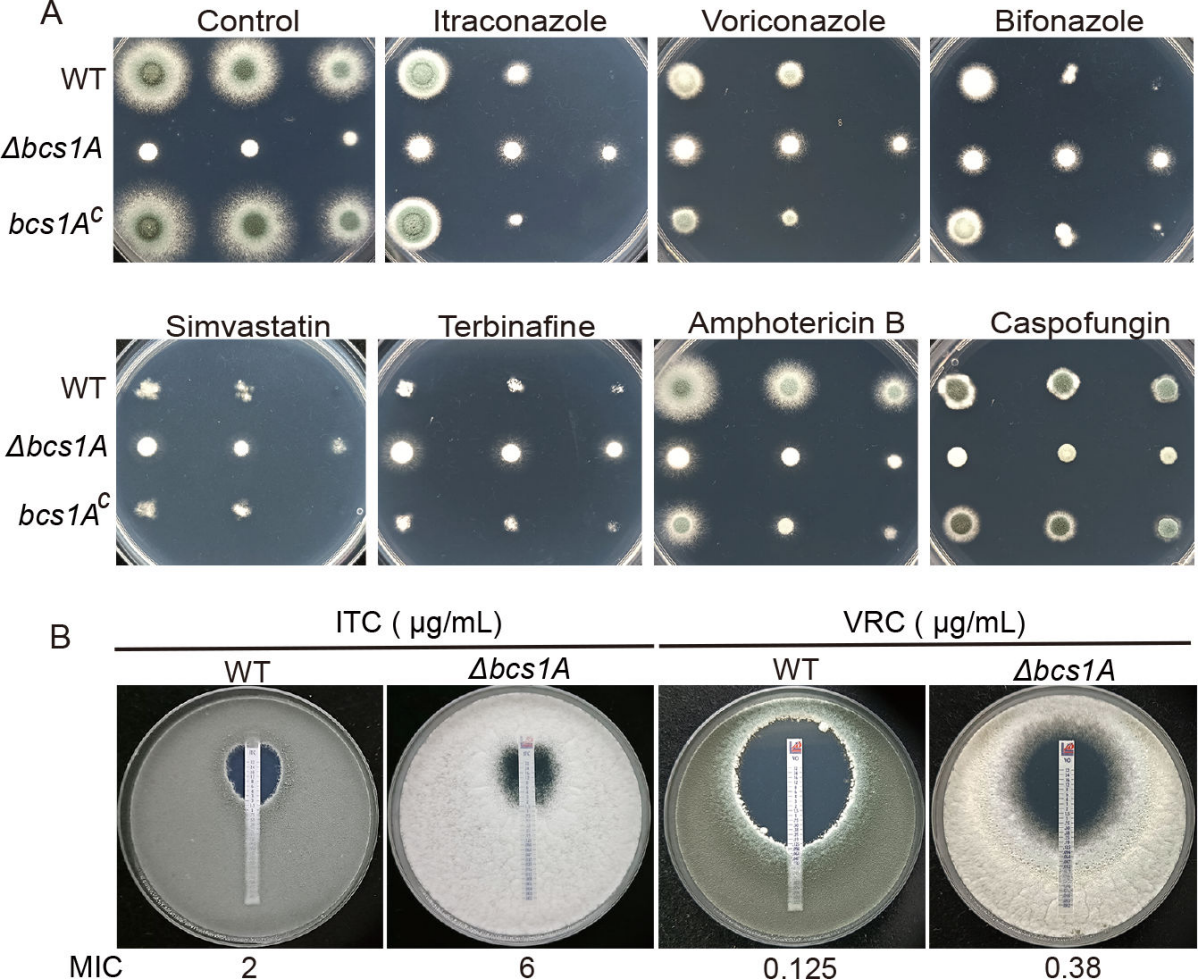
Loss of *bcs1A* reduces susceptibility to antifungal drugs in *A. fumigatus*. (**A**) Colony morphology of the strains after cultivation on minimal medium at 37°C for 2 days, as well as after exposure to various concentrations of antifungal drugs (0.15 µg/mL itraconazole, 0.5 µg/mL voriconazole, 1 µg/mL bifonazole, 0.8 µg/mL terbinafine, 10 µg/mL simvastatin, 1 µg/mL amphotericin B, and 0.8 µg/mL caspofungin) at 37°C for 3 days. (**B**) MIC values of itraconazole and voriconazole were determined using E-test strips on plates containing a mixture of conidia (1 × 10^5^) of the WT or Δ*bcs1A* strains in solid YG medium, followed by incubation at 37°C for 3 days.

As the mitochondria play important roles in the fungal response to antifungal drugs, the optimal functioning of the organelles is crucial. This study examined the phenotypes of Bcs1A-related strains following exposure to media containing various fungicidal agents, including azoles, simvastatin, terbinafine, amphotericin B, and caspofungin. Notably, except for amphotericin B and caspofungin, the Δ*bcs1A* mutant exhibited increased tolerance to voriconazole, itraconazole (ITC), bifonazole, simvastatin, and terbinafine compared with both the parental WT and *bcs1A*-complemented strains (Fig. 3A), suggesting the *bcs1A* plays a role in multidrug tolerance in *A. fumigatus*. To further quantify this tolerance, commercial E‐test strips were then used to measure the minimum inhibitory concentration (MIC) values of Δ*bcs1A* mutant to the azole compounds, such as itraconazole and voriconazole. As depicted in Fig. 3B, the MIC of itraconazole for the Δ*bcs1A* mutant strain was 6 µg/mL, significantly higher than the 2 µg/mL observed for the WT strain. Similarly, the MIC value of voriconazole for the Δ*bcs1A* mutant strain (0.38 µg/mL) was markedly higher than that of the WT strain (0.125 µg/mL). These findings corroborate the critical role of *bcs1A* in modulating multidrug tolerance in *A. fumigatus*.

In yeast, Bcs1 facilitates the assembly of respiratory complex III by transporting and inserting the Rip1 into the mitochondrial membrane. To further determine if the Δ*bcs1A*’s drug-resistant phenotype is caused by the dysfunction of complex III in OXPHOS or it is specific to the loss of function of Bcs1A, the Δ*rip1* deletion mutant was constructed. As shown in Fig. S4, The Δ*rip1* mutant strain exhibited defects in colony growth and conidiation, as well as resistance to the antifungal drugs, consistent with the phenotypes observed in the Δ*bcslA* mutant strain. Therefore, we hypothesize that the drug-resistant phenotype of Δ*bcslA* is attributed to the dysfunction of complex III rather than solely to the loss of Bcs1A function.

### Deletion of *bcs1A* upregulates numerous multidrug tolerance-associated transport genes, and loss of the drug efflux pump *mdr1* decreases azole tolerance in *A. fumigatus*

**Fig 4:**
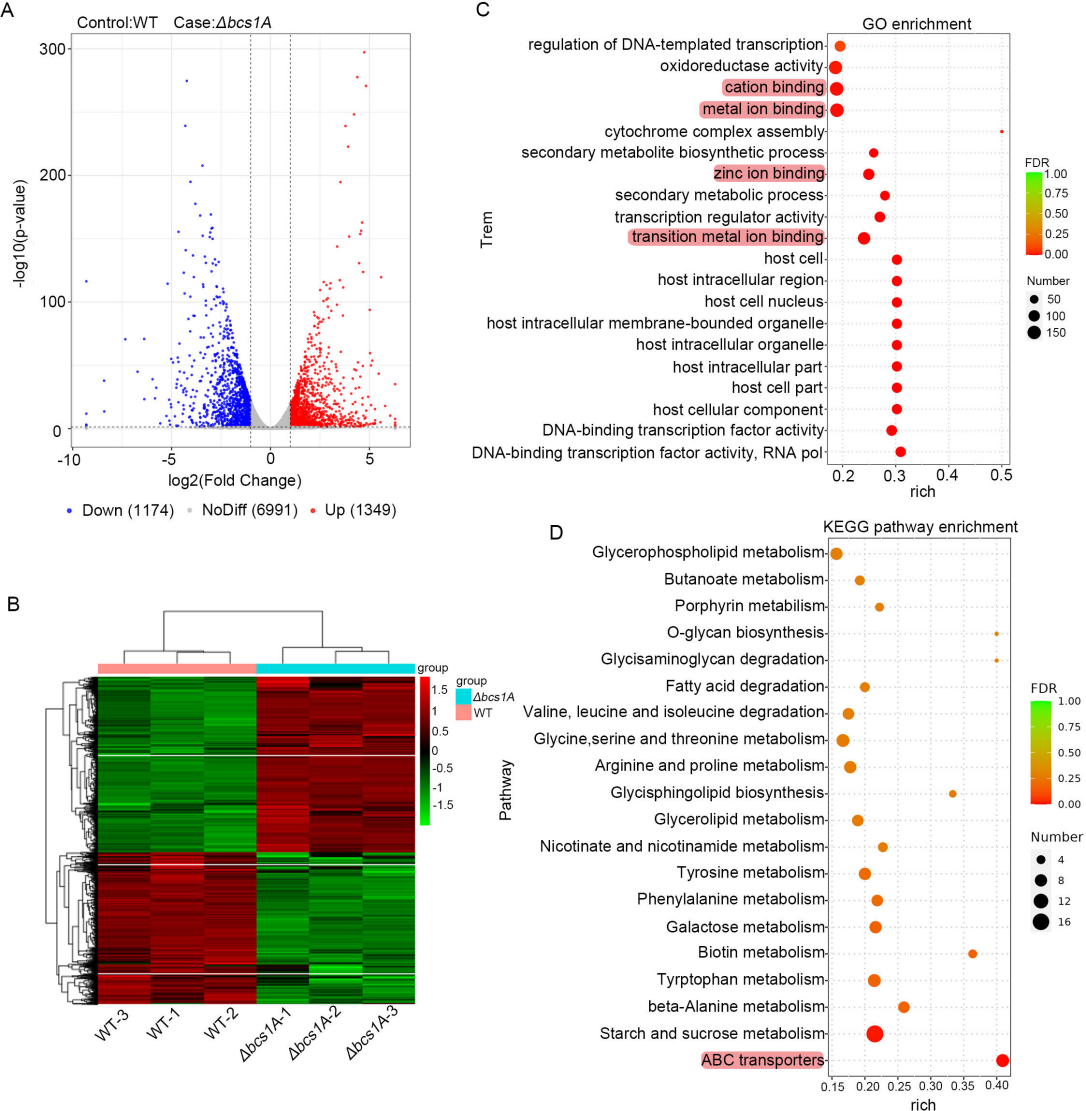
RNA-sequencing analysis of the *bcs1A* mutant and WT strains. (**A**) Volcano plot showing DEGs in the Δ*bcs1A* mutant compared to the WT strain. (**B**). Heatmap comparing the RNA-seq data of the Δ*bcs1A* mutant relative to the WT strain. (**C**) GO analysis of the functional category and (**D**) KEGG analysis of pathway enrichment of DEGs (*P* < 0.05 and lg_2_FC > 1 or < −1).

**Fig 5:**
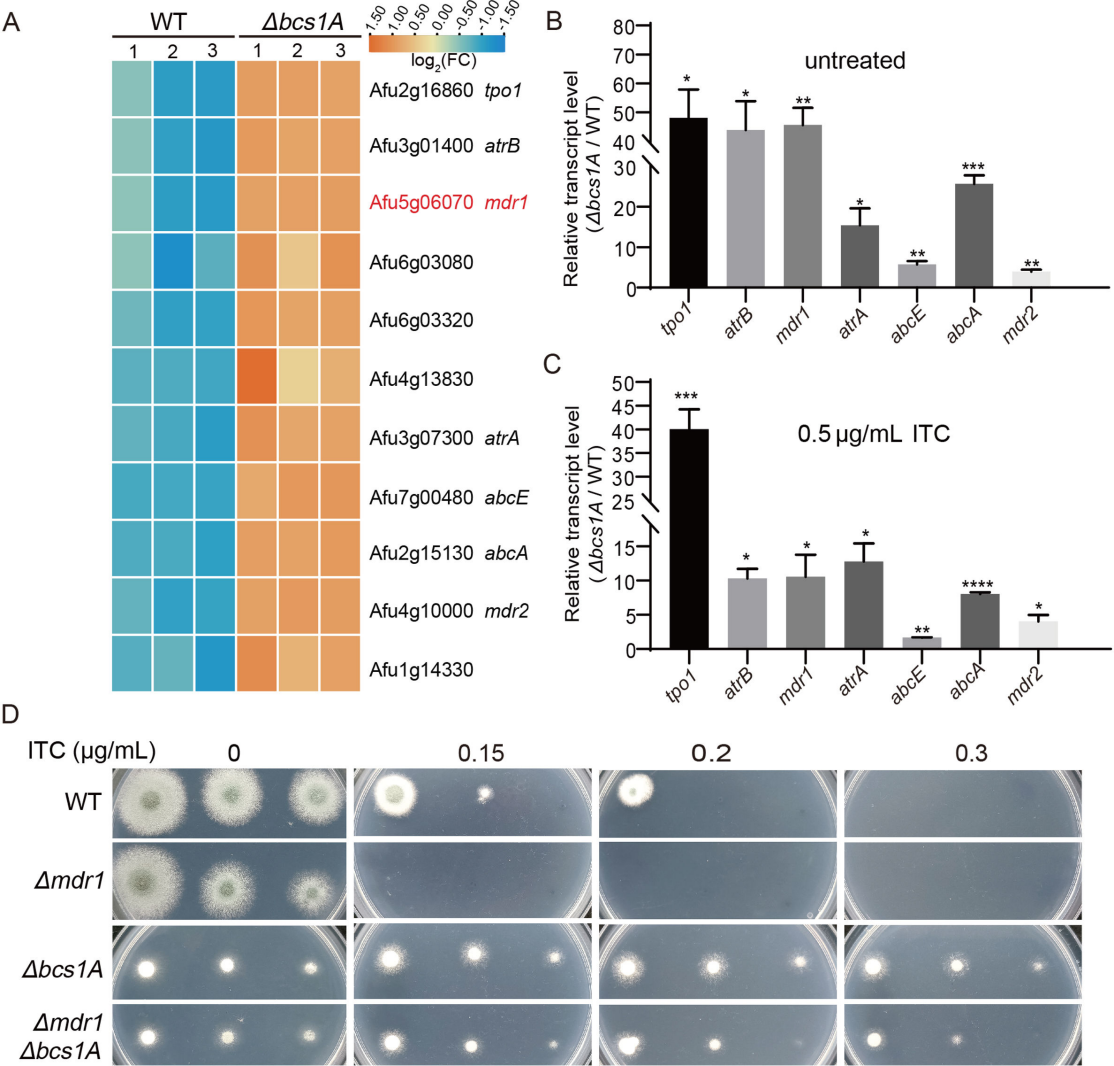
Loss of *bcs1A* leads to upregulating various multidrug resistance-associated transport genes. (**A**) Heatmap showing RNA-seq data of multidrug transport-related genes. Real-time quantitative PCR analysis of indicated genes in the WT and Δ*bcs1A* strains without ITC treatment (**B**) and with ITC treatment for 1 h (**C**). ns, not significant; *, *P* < 0.05; ***, *P* < 0.001; **, *P* < 0.01; ***, *P* < 0.001. (**D**). Colony morphologies of the indicated strains cultured in MM containing different ITC concentrations (0, 0.15, 0.2, 0.3 µg/mL).

To delve deeper into the molecular mechanisms underlying the involvement of Bcs1A in multidrug tolerance, the whole-genome transcriptome profiles of the WT and Δ*bcs1A* strains were analyzed by RNA sequencing (RNA-seq). The total number of reads for each sample is presented in Table S1, with subsequent mapping to the genome database using HISAT2 software (http://ccb.jhu.edu/software/hisat2/index.shtml; Table S2). Normalization of expression levels was performed using fragments per kilobase per million fragments. Identification of differentially expressed genes (DEGs) showed that 1,349 genes were upregulated and 1,174 were downregulated in the Δ*bcs1A* mutant in comparison with the WT strain (Fig. 4A and B; Data set 1), including a series of mitochondrial localization proteins (Data set 1). Furthermore, Gene Ontology (GO) enrichment analysis showed that the upregulated genes involved in multifaceted activities, including GO terms oxidoreductase activity, secondary metabolic processes, and transition metal ion binding, among others (Fig. 4C). Further investigation of pathways associated with the upregulated genes using the Kyoto Encyclopedia of Genes and Genomes (KEGG) database indicated the upregulated genes’ involvement in a variety of metabolic signaling pathways. Specifically, the *bcs1A* deletion mutant showed significant enrichment in pathways associated with ATP-binding cassette (ABC) transporters (Fig. 4D). These are a type of drug efflux pump and include *tpo1*, *mdr2*, *atrB*, *atrA*, *abcE*, *abcA*, and the widely recognized drug efflux pump *mdr1* (Fig. 5A).

To confirm the RNA-seq findings, quantitative real-time PCR (qRT-PCR) was conducted using biological replicate samples from both the Δ*bcs1A* mutant and the WT control. As depicted in Fig. 5B, the results indicated varying degrees of upregulation of the aforementioned transport genes in the Δ*bcs1A* strain relative to the WT control. The expression of transport genes is critical for fungal adaptation to environments containing drugs; hence, the mRNA levels of the ABC transporters in the WT and Δ*bcs1A* mutant strains were measured by qRT-PCR after the treatment with ITC. Likewise, compared to the WT, the selected genes showed significant upregulation in the Δ*bcs1A* mutant (Fig. 5C), implying that Bcs1A may be involved in drug susceptibility by affecting the expression of drug transporters. To further verify whether the multidrug resistance of the Δ*bcs1A* mutant is caused by the upregulation of efflux pumps, the gene *mdr1*, a key drug efflux pump in fungi, was deleted in both the WT and Δ*bcs1A* mutant. As shown in Fig. 5D, the double *bcs1A* and *mdr1* mutants exhibited increased susceptibility compared to the single *bcs1A* mutant when treated with the antifungal drug ITC. Taken together, these data suggest that the *bcs1A* may contribute to multidrug tolerance by affecting the transcriptional levels of efflux pumps.

### Bcs1A regulates metal ion homeostasis in *A. fumigatus*

**Fig 6:**
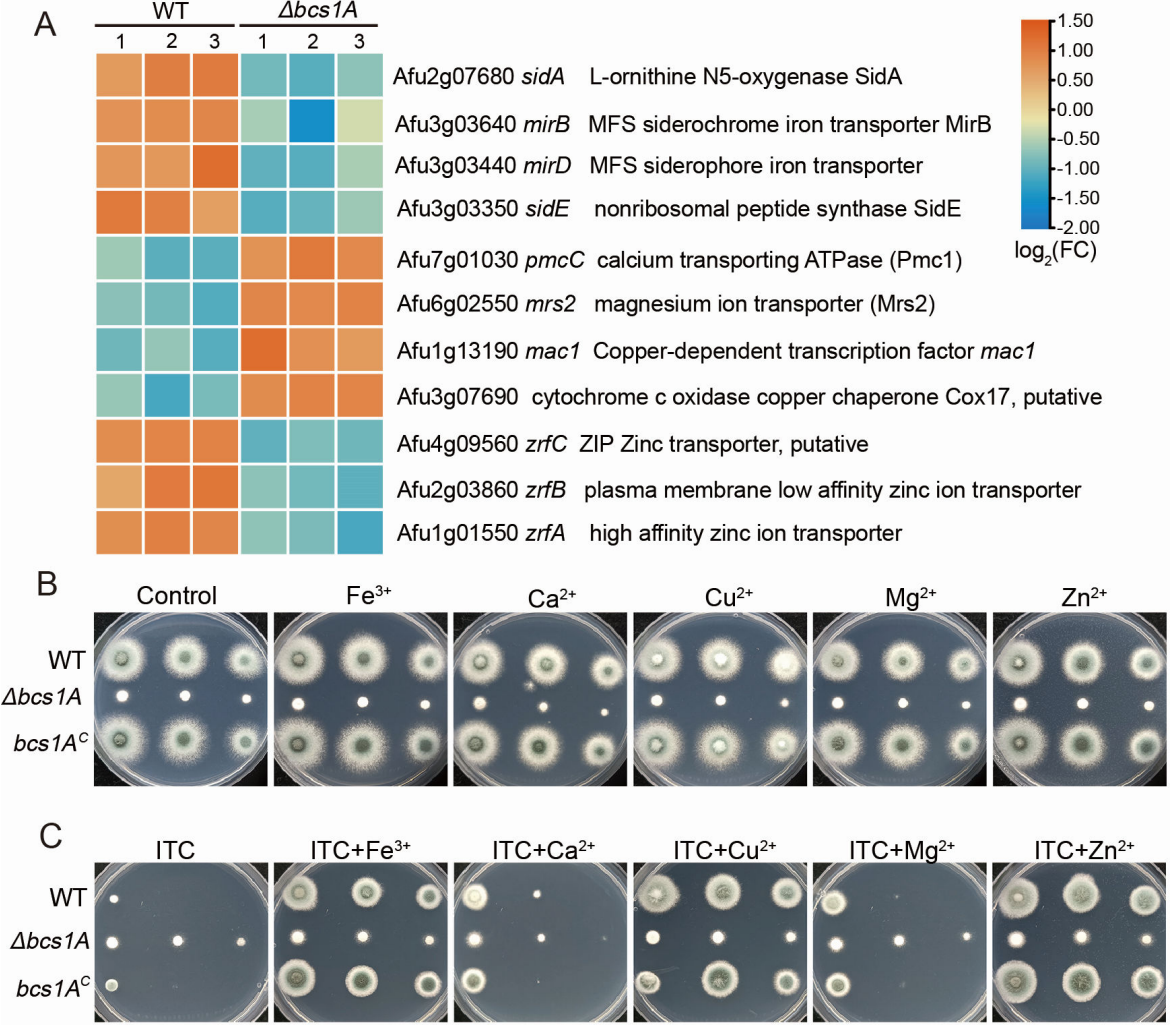
Addition of metal ions can increase azole tolerance of *A. fumigatus*. (**A**) Heatmap showing metal ion transport-related genes in the Δ*bcs1A* mutant and its parental WT strain. (**B**) Colony morphologies of strains grown on MM in the presence of various metal ions (FeCl_3_ 1 mM; CaCl_2_ 50 mM; CuSO_4_ 100 µM, MgCl_2_ 50 mM; ZnCl_2_ 1 mM) at 37°C for 2 days or (**C**) with 0.2 µg/mL ITC at 37°C for 2.5 days.

The RNA-seq analysis demonstrated that the transcriptional levels of genes associated with metal ion transport, including iron, calcium, magnesium, copper, and zinc transporters, were affected in the *bcs1A* deletion mutant compared to the WT control (Fig. 4C and 6A). To confirm these findings, qRT-PCR was performed on 11 selected genes, which demonstrated that seven genes (*sidA, mirB*, *mirD*, *sidE*, *zrfA*, *zrfB*, *zrfC*) were downregulated, while four genes (*pmcC*, *mrs2*, *mac1*, Afu3g07860) were upregulated (Fig. S4). These results suggest that Bcs1A plays an important role in maintaining metal ion homeostasis in *A. fumigatus*. Considering the well-documented correlation between intracellular metal ion homeostasis and the fungal reaction to antifungal medication (39). It was hypothesized that disrupted metal ion regulation might contribute to the observed multidrug resistance shown by the Δ*bcs1A* mutant. The WT, Δ*bcs1A* mutant, and *bcs1A* complementary strains were then cultured on media containing different metal ions with and without the addition of ITC. As shown in Fig. 6B, metal ion supplementation did not affect colony growth among the *bcs1A*-related strains under normal conditions. However, when cultured with ITC, the addition of metal ions could ameliorate the drug-induced growth defects in the WT and *bcs1A* complementary strains (Fig. 6C), suggesting that metal ion homeostasis is linked to antifungal drug resistance, potentially mediating the enhanced multidrug resistance phenotype seen in the Δ*bcs1A* mutant.

## DISCUSSION

Mitochondria are commonly referred to as the powerhouses of the eukaryotic cell, underpinning numerous critical biological processes (34, 36, 43). Previous studies highlight the essential role of Bcs1 orthologs in mitochondrial function, primarily through facilitating the transport and integration of the folded Rip1 into developing complex III of the mitochondrial respiratory chain (46, 47, 49).

Bcs1 was the first identified chaperone in yeast (48, 50). Subsequently, six Bcs domain proteins (Bbbcs1a–f) were identified, while only Bbbcs1c is considered the ortholog of yeast bcs1 in the filamentous entomopathogenic fungus *B. bassiana* (53). The findings of the present study showed that Bcs1A is a homolog of the yeast Bcs1 and contributes to proper mitochondrial function in the filamentous fungal pathogen *A. fumigatus* (Fig. 1A and 2). Consistent with previous reports on the unicellular yeast and multicellular *B. bassiana*, analysis of subcellular localization showed that the Bcs1A was located in the mitochondria of *A. fumigatus* (Fig. 1B). Further bioinformatic analysis revealed that Bcs1 orthologs are widely distributed in eukaryotes, and the two domains (BCS1_N and AAA) are contained in all selected Bcs1 homologs, suggesting the strong conservation of Bcs1 as a mitochondrial protein from fungi to *Homo sapiens* (Fig. 1A). Phenotypic analysis showed serious impairment of the growth of the Δ*bcs1A* strain compared with the WT and complemented strains (Fig. 1C and D), suggesting Bcs1A is the *A. fumigatus* homolog of the yeast Bcs1. Further functional analysis showed that a series of mitochondria-related functions, including carbon source metabolism, the generation of ROS and ATP, the MMP, and osmosensing, were abnormal in the Δ*bcs1A* mutant strain, indicating that Bcs1A is critical for proper mitochondrial function in *A. fumigatus* (Fig. 2).

Numerous studies have reported that impaired vegetative growth caused by dysfunctional mitochondria is an adaptive strategy used by fungi to counteract the presence of harmful antifungal drugs (29, 39). The phenotypic analysis conducted in this study revealed that the Δ*bcs1A* mutant strain displayed decreased susceptibility to antifungal drugs such as azoles, statin, and allylamine (Fig. 3A). The further results of the MIC test were another demonstration that Bcs1A is critical for the fungal response to azoles (Fig. 3B). The antifungal mechanisms of these drugs have been extensively studied. Azoles inhibit the synthesis of the cell membrane lipid ergosterol by targeting lanosterol demethylation (Cyp51/Erg11), resulting in the accumulation of toxic intermediate metabolites and ultimately cell death (19). Statins and allylamines have fungicidal activity by targeting 3-Hydroxy-3-methylglutaryl coenzyme A (HMG CoA) reductase and squalene epoxidase, two key enzymes in the ergosterol biosynthesis pathway (56, 57). Thus, ergosterol synthesis may underlie the Bcs1A-mediated response to drugs. However, there was no detective difference between the Δ*bcs1A* and the WT strains when grown with amphotericin B that induces fungicidal effects by direct binding to ergosterol in the cell membrane (Fig. 3A), resulting in membrane permeabilization and cell death, suggesting the mechanism of Bcs1A-mediated drug tolerance is multifaceted.

In yeast, mitochondrial dysfunction can lead to azole resistance by activating the pleiotropic drug resistance pathway, a direct regulator of the expression of drug efflux pumps (29, 35). RNA-seq and qRT-PCR results revealed an upregulation of multiple efflux pumps in the Δ*bcs1A* mutant (Fig. 4D, 5A through C). Subsequent confirmatory experiments involving the deletion of *mdr1* showed a partial reduction in azole tolerance (Fig. 5D). Therefore, it is postulated that the increased expression of drug efflux pumps resulting from mitochondrial dysfunction may be the primary factor contributing to drug resistance in the Δ*bcs1A* mutant. Recent studies have shown a close association between mitochondrial metal ion homeostasis and drug efflux pump expression (39, 58). For example, intracellular calcium disorder induced by mitochondrial dysfunction causes persistent activation of the zinc finger transcription factor CrzA, triggering a global overexpression of multidrug transport genes (39). Both RNA-seq and RT-qPCR showed the abnormal expression of metal ion-related genes in the Δ*bcs1A* mutant (Fig. 4C and 6A). Subsequent phenotypic analysis showed that the WT strain exhibited increased tolerance to itraconazole in media supplemented with various metal ions (FeCl_3_, CaCl_2_, CuSO_4_, MgCl_2_, ZnCl_2_) (Fig. 6B and C), suggesting that metal ion homeostasis may be another reason for the increased azole tolerance in the Δ*bcs1A* mutant. Besides, the activity of energy-dependent efflux pumps is crucial for drug tolerance in fungi (59). Hence, the abnormal ATP generation may contribute to drug tolerance by improving the activities of drug efflux pumps and increasing the efflux of intracellular drugs in the Δ*bcs1A* mutant.

Numerous studies have demonstrated that the components of respiratory chain complex III, Bcs1 included, are excellent potential drug targets as they are easily targeted by many natural antibiotics (49, 60). Defective BCS1L (BCS1-like), the human homolog of the yeast Bcs1, may cause various complex III-related pathologies, such as neonatal proximal tubulopathy, hepatic involvement, and encephalopathy (52). In agreement with previous studies, the bioinformatic analysis showed conservation of Bcs1 mitochondrial proteins from fungi to humans (Fig. 1A). Hence, drugs that bind directly to Bcs1A may have side effects when used for the clinical treatment of aspergillosis. Of note, it is reported that the use of pentamidine and clarithromycin, two FDA-approved drugs, can partially rescue the *bcs1* mutant in yeast (41). There is thus a possibility that the use of pentamidine or clarithromycin together with antifungal drugs may represent a novel treatment strategy for aspergillosis caused by Bcs1A-mediated (mitochondrial dysfunction) drug resistance.

Collectively, our research underscores the critical role of the mitochondrial protein Bcs1A in modulating antifungal drug responses through the regulation of drug efflux pumps. Nevertheless, the widespread conservation of Bcs1A among different species highlights its importance, which may hinder its potential as a viable drug target in humans due to the likelihood of adverse effects.

## MATERIALS AND METHODS

### Strains, media, and culture conditions

The *A. fumigatus* strains utilized in this study are detailed in Table 1. The culture media employed encompassed MM consisting of 50 mL/L 20 × salt solution, 1% glucose, 1 mL/L trace elements, and 2% agar, with a pH of 6.5, as well as YG medium containing 0.5% yeast extract, 2% glucose, 1 mL/L trace elements, and 2% agar (61). Agar was excluded from all liquid media utilized. The glycerol or ethanol medium mirrored the glucose medium, except for substituting glycerol or ethanol as the carbon source. Cultivation of all strains occurred at 37°C for the specified durations.

**TABLE 1:** Strains used in this study

Strain	Genotype	Source
A1160	Δ*ku80, pyrG*	LL-lab
WT	Δ*ku80, A1160::pyrG*	LL-lab
Bcs1A-GFP	*∆ku80, pyrG, bcs1A::gfp::pyrG*	This study
Δ*bcs1A*	Δ*ku80, pyrG,* Δ*bcs1A::pyrG*	This study
*bcs1A^C^*	Δ*ku80, pyrG,* Δ*bcs1A::pyrG, bcs1A::bcs1A::hph*	This study
Δ*mdr1*	*∆ku80, pyrG,* Δ*mdr1::hph*	This study
Δ*mdr1*Δ*bcs1A*	Δ*ku80, pyrG,* Δ*bcs1A::pyrG,* Δ*mdr1::hph*	This study
Δ*rip1*	Δ*ku80, pyrG,* Δ*rip1::pyrG*	This study

### Deletion and complementation of *bcs1A*

All primers used in this study are displayed in Table 2. For the construction of the *bcs1A* deletion strain, the open reading frame (ORF) of *bcs1A* was substituted with the selective marker *pyrG*. The *pyrG* marker was amplified from the plasmid pXDRFP4 using the primer pair PyrG-F/PyrG-R. The approximately 1.5 kb of the upstream and downstream flanking sequences was amplified by using the primer pairs bcs1A-P1/P3 as well as bcs1A-P4/P6, respectively. The abovementioned three PCR products were purified and then employed as templates to generate the *bcs1A* deletion cassette by using the primer pair bcs1A-P2/P5. The resulting fusion product was subsequently used to transform the recipient strain A1160 (62).

**TABLE 2:** Primers used in this study

Name	Sequence (5´ to 3´)
bcs1A-gfp-p1	GGGTGTGTAAGACGCGTCTG
bcs1A-gfp-p3	CCAGCGCCTGCACCAGCTCCTGGTCGCCCTCGGCTGAATCAT
bcs1A-gfp-p4	CATCAGTGCCTCCTCTCAGACAGGTATGTACATTATCATGACGTG
bcs1A-gfp-p6	AGGAATAGTGTACCTCAAG
bcs1A-gfp-p2	TACATACTCACTTTGCACTGG
bcs1A-gfp-p5	CTTTTGATGAGAGCCTACCTG
gfp-pyrG F	GGAGCTGGTGCAGGCGCTGG
gfp-pyrG R	CTGTCTGAGAGGAGGCACTGATG
bcs1A-p1	CCGCACGGTATCCAGTATCTC
bcs1A-p3	CCTCTAGATGCATGCTCGAGC CCCAGTCTACGGAGATGAGC
bcs1A-p4	CATCAGTGCCTCCTCTCAGACAG TATGTACATTATCATGACGTGT
bcs1A-p6	GAATAGTAACCTATCGGGATG
bcs1A-p2	CATATTCTTAATTCCCGAGC
bcs1A-p5	TTGAGGGTAGTACTGCTGTT
PyrG-F	GCCTCAAACAATGCTCTTCACC
PyrG-R	CTGTCTGAGAGGAGGCCTGATG
bcs1A-self-F	CAAGGACGATTCTTATCCAT
bcs1A-self-R	AGGAAAATGATACGCTCCTC
Com-bcs1A-F	ACTGCAGGCATGCAAGCTT AATTTCTTCGCAGGTTCGAG
Com-bcs1A-R	CGACGGCCAGTGCCAAGCTTCCAACATTGACATATACCCCTC
RT-tubA F	TTCCGTCCCGACAACTTCGT
RT-tubA R	TCACAGCCTTCAGCCTCACG
RT-tpo1 F	CTGCGCCCTATTCAAATGCTT
RT-tpo1 R	GAAAAGGTACAGGATTCCGTACACCA
RT-mdr1 F	GCTCTGCCTGAAGGTTACG
RT-mdr1 R	TGTGGGCTGTTTTGATTGT
RT-atrB F	CTGGCCTCGACGGTCAATCC
RT-atrB R	TTGGCCAACAGCAACAGGGT
RT-abcA F	CGGGCTTTTGGATTTTCATGTACC
RT-abcA R	TCAATATCTGAGCACTTGACGCTGG
RT-atrA F	GCATCCACGAGTCCAAGCGA
RT-atrA R	CCGCGCATATGCCAAGCATC
RT-abcE F	GCCACCGACCCAAAGCAGGT
RT-abcE R	TGTGCATGGTAAGGCGGCAA
RT-mdr2 F	AGATCCGATACGATTATTGTCC
RT-mdr2 R	TGCCACTCCATCAATTTGGT
RT-sidA F	AACACTTGAGACCTACGGGACA
RT-sidA R	TTACAACCTTGAAGCCAGATGC
RT-mirB F	AGCTTGATCTATTCGTCCCTCC
RT-mirB R	CCCTTGGTCTGAGTCATGTTTT
RT-mirD F	ACTACTACACATCCTACCTGC
RT-mirD R	AATAGCCAGACTCCCGAGA
RT-sidE F	AATGTTCCTGTGGACCGCTTC
RT-sidE R	GGCGCTTCTCAGATTGCTTC
RT-pmcC F	ACACCGTCATCTTCAACACC
RT-pmcC R	GCGTTGATCACCATGAACCA
RT-mrs2 F	ACCAAGAGCCGTCCACTCCT
RT-mrs2 R	GTAGCCGTGGCTCGTTCGTT
RT-mac1 F	TGACAACCCTGATGCCACT
RT-mac1 R	AGCTGCCATTTGGACT
RT-3g07690 F	TCTGTAAGACCGAGAAGTCC
RT-3g07690 R	AACCTTGAATCCGTATCCAG
RT-zrfC F	TGTCTTCCACCAGTTCTTCGAGG
RT-zrfC R	ATCACGGCAAAGGTCCC
RT-zrfB F	TTCATCCATCTTTTGGGACCG
RT-zrfB R	GAACAGCACAACAATGGTCA
RT-zrfA F	CGCATCTCCTCCATCTTCGT
RT-zrfA R	CAAAGTATCGCCCGAACAGGT
Rip1-P1	CACCAGCAACAGCACAAACC
Rip1-P2	CGTGTGAACCAGAGCTTTGTC
Rip1-P3	CGATTAAGTTGGGTAACGCCAGGCTGGGGCCCACGACAATTC
Rip1-P4	ATAAGTAGCCAGTTCCCGAAAGCCTCTTCGTAAGTCGCTAGATG
Rip1-P5	CTGCTCTTACATCCACATCG
Rip1-P6	GTCGCAAACGACTCGTGAAG
Rip1-up	GCTCTCTTCCCCCCTCAACT
Rip1-down	ACGGGCTTTCCACGCCACTT

The transformants were grown on MM and verified by diagnostic PCR using primers bcs1A-P1/PyrG-R, bcs1A1-self-F/R, and PyrG-F/bcs1A-P6, respectively. For complementation of the Δ*bcs1A* mutant, the *bcs1A-*complemented fragment was amplified using the primer pair Com-bcs1A-F/R with the wild-type genomic DNA as a template, which contains the native promoter sequence, the complete ORF, and the termination sequence of *bcs1A*. This fragment was then cloned into the Hind III site of the plasmid *pAN7‐1* and then transformed into the Δ*bcs1A* strain.

### Construction of Bcs1A-GFP strain

To label Bcs1A with a GFP tag at the C terminus, we first amplified the upstream sequence (without the termination codon) and downstream sequence of *bcs1A* from A1160 strain gDNA using the primers bcs1A-gfp-p1/p3 and bcs1A-gfp-p4/p6, respectively. Then, a GFP‐pyrG fragment was amplified from the plasmid pFNO3 using primer pair gfp-pyrG F/R. The abovementioned three DNA fragments were then employed as templates to generate the Bcs1A-gfp cassette using the primers bcs1A-gfp-P2/p5. The fusion production was then transformed to A1160 strain and transformants were detected by diagnostic PCR using the primer pairs bcs1A-gfp-p1/gfp-pyrG R and gfp-pyrG F/bcs1A-gfp-p6.

### Fluorescence microscopy

To visualize the subcellular distribution of Bcs1A, the GFP-labeled strain was incubated in 500 µL of liquid MM on glass coverslips for a duration of 12 h. Samples were fixed in 4% formaldehyde for 30 min and then incubated in the dark with the mitochondrial dye MitoTracker Red CMXRos (Beyotime) for 5 min used at a final concentration of 5 nM. Images were obtained by using an Olympus BX53 microscope (Tokyo, Japan).

### Flow cytometry

The accumulation of Rh123 was carried out to determine mitochondrial membrane potential in *A. fumigatus* as described previously (63). Briefly, a total of 5 × 10^6^ spores of the related strains were incubated in 1.5 mL of liquid YG medium at 37°C until conidia began to germinate. The samples were then washed three times with phosphate-buffered saline (PBS) and incubated with Rh123 (Beyotime) at a final concentration of 10 µM for 30 min in the dark, and then washed three times with PBS to remove Rh123. Fluorescence intensity was measured by flow cytometry (BD FACSVerse, USA).

### Detection of reactive oxygen species

To measure the production of ROS, 1 × 10^7^ spores of the related strains were inoculated into 100 mL of liquid MM at 37°C, 220 rpm, until conidia grew into mycelia. Then, 2´,7´-dichlorodihydrofluorescein diacetate (MedChemExpress) was added to the medium at a final concentration of 15 µM and incubated at 37°C for 1.5 h in the dark. The collected mycelia were pulverized with liquid nitrogen and subjected to incubation in PBS. The supernatant was next collected by centrifugation at 12,000 × *g* and 10 min at 4°C. Fluorescence intensity was measured with an excitation wavelength of 504 nm and an emission wavelength of 524 nm using PerkinElmer EnSight (USA). The relative production of ROS was normalized by the fluorescence intensity to the total protein concentration, which was measured by a Bicinchoninic Acid Assay (BCA) protein assay kit (Beyotime, China).

### RNA-seq and qRT-PCR

For RNA-seq, 1×10^8^ conidia of relative stains were inoculated into liquid MM in a rotary shaker at 37°C, 220 rpm, 18 h. Then, the mycelium was collected and frozen with liquid nitrogen. Three biological replicates of the WT and Δbcs1A samples were subsequently submitted to Personal Biotechnology (Nanjing, China) for transcriptome analysis using the Illumina platform. The raw data have been submitted to SRA (https://www.ncbi.nlm.nih.gov/sra) at NCBI with six accession numbers SRR28521163–SRR19440565 (WT-1, WT-2, and WT-3, three biological replicate samples of WT) and SRR28521166–SRR19440568 (Δbcs1A-1, Δbcs1A -2, and Δbcs1A -3, three biological replicate samples of Δbcs1A).

For qRT-PCR, samples were prepared as aforementioned. Total RNA was isolated using a UNIQ-10 column total RNA purification kit (Shanghai Sangon Biotech, Shanghai, China) and cDNA was generated using a HiScript II Q RT SuperMix for qPCR kit (Vazyme) according to the manufacturer’s instructions. Quantitative PCR was performed with AceQ qPCR SYBR Green Master Mix (Vazyme) by LightCycler 480 (Roche). Results were normalized to *tubA* expression, and expression levels were calculated using the 2^-∆∆CT^ method.

## SUPPLEMENTAL MATERIAL

Supplemental figures and tables spectrum.01172-24-s0001.pdf Fig. S1-S5; Tables S1 and S2.

Supplemental material spectrum.01172-24-s0002.xlsx Different expression genes of RNA-Seq.

ASM does not own the copyrights to Supplemental Material that may be linked to, or accessed through, an article. The authors have granted ASM a non-exclusive, world-wide license to publish the Supplemental Material files. Please contact the corresponding author directly for reuse.
