# Supplementary material for: The mitochondrial protein Bcs1A regulates antifungal drug tolerance by affecting efflux pump expression in the filamentous pathogenic fungus Aspergillus fumigatus
Source: Microbiol Spectr. 2024 Aug 20;12(10):e01172-24. doi: 10.1128/spectrum.01172-24 (PMC11448404; doi:10.1128/spectrum.01172-24)
Supplement: Supplemental figures and tables — Fig. S1-S5; Tables S1 and S2. [file spectrum.01172-24-s0001.pdf]

Supplementary Information

The mitochondrial protein Bcs1A regulates antifungal drug tolerance by affecting efflux pump expression in the filamentous pathogenic fungus *Aspergillus fumigatus*

Guorong Yang[1], Weiwei Shi[2], Wenlin He[1], Jing Wu[1], Sutao Huang[3], Li Mo[4], Junjie Zhang[4], Huaxue Wang[2], Xiaogang Zhou[1]

1 Anhui Key Laboratory of Infection and Immunity, School of Basic Medicine, Bengbu Medical University, Bengbu, China.

2 Departments of Critical Care Medicine, The First Affiliated Hospital of Bengbu Medical University, Bengbu, China.

3 School of Life Sciences, Bengbu Medical University, Bengbu, China

4 School of Fundamental Sciences, Bengbu Medical University, Bengbu, China

Address correspondence to Xiaogang Zhou, Zhouxg@bbmc.edu.cn, or Huaxue Wang, huaxuew2010@163.com

Guorong Yang, Weiwei Shi and Wenlin He contributed equally to this work.

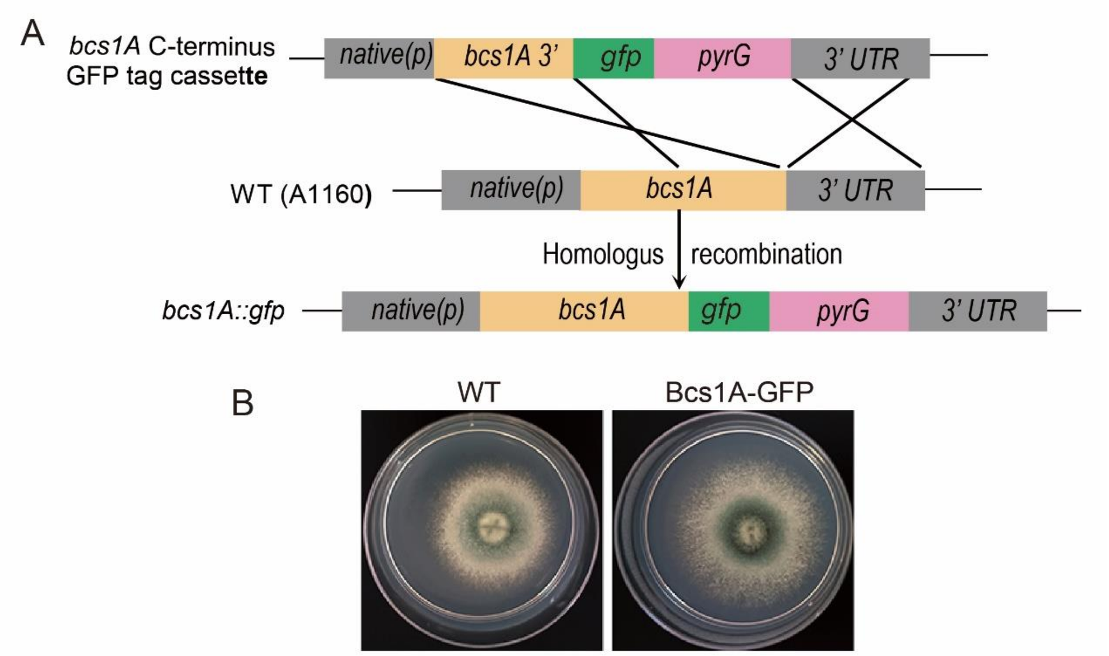

Fig S1. Generation of the Bcs1A-GFP strain. (A). The diagram illustrates the strategy for the construction of the Bcs1A-GFP strain. (B).The phenotype of the WT and Bcs1A-GFP strains cultured in MM at 37◦C for 2 days.

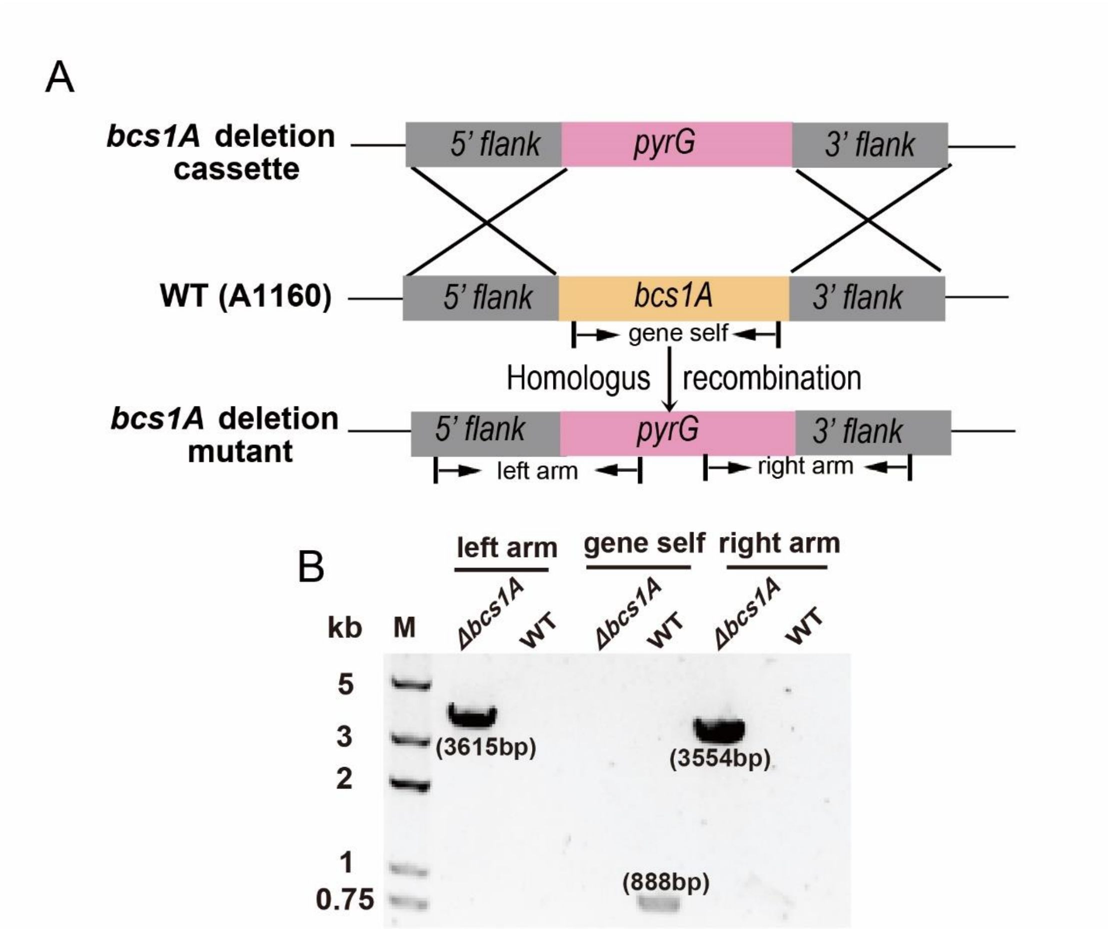

Fig S2. Generation of the *bcs1A* deletion strain. (A). Schematic view of *bcs1A* full-length deletion using homologous recombination strategy. (B). Diagnostic PCR confirmed the successful construction of the *bcs1A* deletion strain.

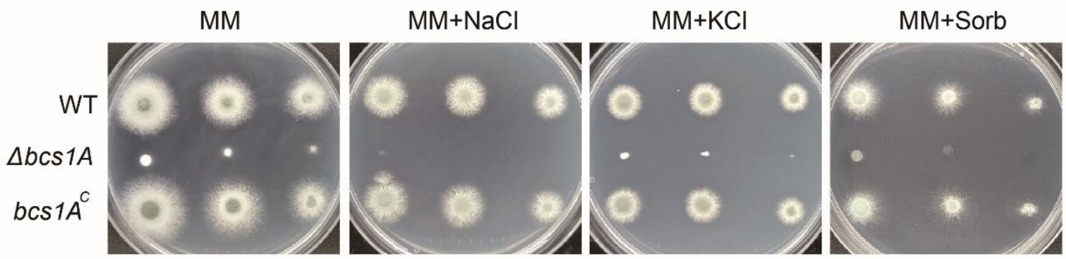

Fig S3. Colony morphologies of WT, *Δbcs1A,* and *bcs1A*^{C} strains cultured in MM supply with or without 1 M NaCl, 1 M KCl, and 1.2 M sorbitol, respectively.

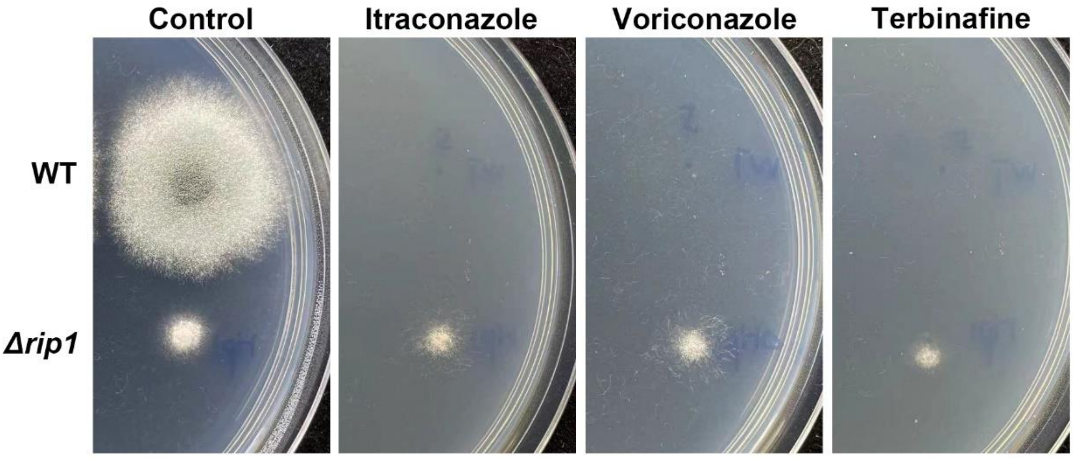

Fig S4. Loss of *rip1* reduces susceptibility to antifungal drugs in *A. fumigatus*. Colony morphology of the strains after cultivation on minimal medium or exposure to various concentrations of antifungal drugs (0.15 μg/mL itraconazole, 0.5 μg/mL voriconazole, 0.8 μg/mL terbinafine) at 37°C for 3 days.

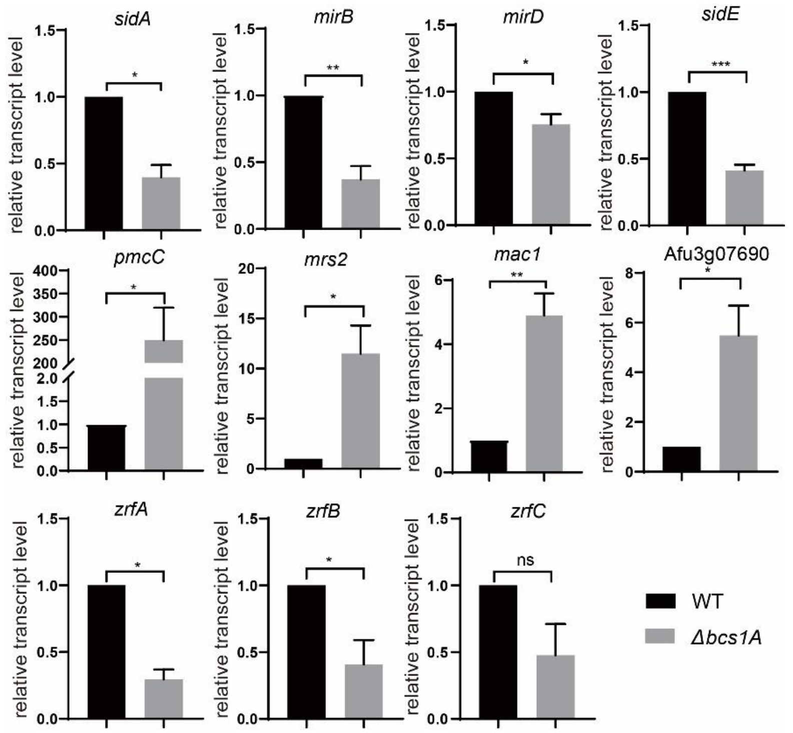

Fig S5. Quantitative RT-PCR comparison of metal ion-related genes between the WT and *Δbcs1A* mutant strains. ns, not significant， *,$P < 0.05$, **, $P < 0.01$, ***, $P < 0.001$.

Supplement table 1: Disembarking data information

Sample	Reads No.	Bases (bp)	Q30 (bp)	N (%)	Q20 (%)	Q30 (%)
bcsA_1	40277442	6081893742	5736510197	0.000664	98.09	94.32
bcsA_2	41311714	6238068814	5843079122	0.000659	97.83	93.66
bcsA_3	42112582	6358999882	5959222604	0.000665	97.85	93.71
WT_1	40381004	6097531604	5717249601	0.000668	97.87	93.76
WT_2	40441908	6106728108	5722495967	0.000658	97.84	93.7
WT_3	43774354	6609927454	6207555484	0.000624	97.93	93.91

Reads No.: total reads nouber
Bases (bp): total bases number
Q30(bp):The total number of bases with a base identification accuracy of 99.9% or more
N(%):Fuzzy base percentage
Q20(%):Percentage of bases with a base recognition accuracy of more than 99%
Q30(%):Percentage of bases with a base recognition accuracy of more than 99.9%

Supplement table 2: RNA-Seq map information

Sample	Clean_Reads	Total_Mapped	Multiple_Mapped	Uniquely_Mapped
bcsA_1	38277764	36964468 (96.57%)	355441 (0.96%)	36609027 (99.04%)
bcsA_2	39242970	37802453 (96.33%)	372845 (0.99%)	37429608 (99.01%)
bcsA_3	40015576	38592017 (96.44%)	360114 (0.93%)	38231903 (99.07%)
WT_1	38292452	36705746 (95.86%)	772877 (2.11%)	35932869 (97.89%)
WT_2	38341164	36759019 (95.87%)	783582 (2.13%)	35975437 (97.87%)
WT_3	41519268	39691135 (95.60%)	937166 (2.36%)	38753969 (97.64%)

Clean Reads:total number of sequences used for alignment
Total Mapped: compare the total number of sequences in the reference genome
Muitiple Mapped:the total number of sequences compared to multiple locations
Uniquely Mapped: the total number of sequences matched to only one location
